# Foreign

**Published:** 1840-09-05

**Authors:** 


### FOREIGN.

*Dr. Macartney on the cure of Wounds without Inflammation.**—A sufficient time has now elapsed, since the publication of my treatise on Inflammation, to enable me to ascertain, through various channels, the manner in which that work has been received by the profession and the public. I have the satisfaction of knowing that both the principles and the practice advocated by me, have been adopted, and acted on, to the fullest extent, by those practitioners whose knowledge of the animal economy I the most respect; and that all the individuals *not* of the profession, who have experienced, in their own persons, the possibility of wounds being healed without inflammation, have the most perfect confidence in the treatment I have recommended, and are fully competent to carry it into effect. I have also discovered that many surgeons, without acknowledging the source from whence they derive their information, are adopting the remedial means by degrees; but in consequence of their not making themselves acquainted with the principles which could alone direct them aright, they commit many mistakes; for instance, in proposing to use the water dressing, the wet lint has been left without any covering, and thus, in a few hours, was converted into a dry application. In other cases, I have seen the injured limb in a depending position, or tightly bound up, or used in its accustomed movements; and, in two or three instances, I have known the oiled silk to have been placed next the skin, and the wet lint on the outside of it, that evaporation might not be impeded, as was said.

There is another class of practitioners, that I am happy to say is not numerous, who defend the hitherto universal opinion of inflammation being salutary, in all cases of open wounds more especially, although the arguments they employ are so futile, that one would be almost tempted to suppose that these adversaries are friends in disguise. It is necessary, however, for me to point out the invalidity of the objections which have been made to my theory respecting inflammation; and, in doing so, I shall omit the names of individuals, in order to avoid personal altercation, which would be unworthy of those who have to discuss a subject of such high importance to mankind.

1st. The main question at issue, and to which I shall confine myself at present, is, can inflammation ever be necessary, or even accessory, to the reparative and curative processes? As the negative reply to this question, it has been asserted, 1st, that an exalted, or irregularly-excited state of the vital powers is essential to reparative action, and that such excited condition only differs in degree from the most severe inflammation. I, however, deny that an increased sensibility and unusual dilatation of the arteries are necessary, or even desirable, in the healing of common wounds. The afflux of blood to a part, is in proportion to the quantity of new material required, and not in relation to the amount or nature of the injury sustained; and hence we find it most remarkable in certain natural and healthy processes of reparation, such as the growth of the new horns in the males of the deer kind, and also in the provisions made for the attachment and nourishment of the fœtus in the viviparous animals; and again, a mere augmentation of sensibility and a determination of blood, should not be confounded with inflammation, between which and them there is an essential difference, the former being characterized by the peculiar pain, the injurious effusions, and the sympathetic irritation of the constitution.

2dly. Some who assume the necessity of inflammation, have gone so far as to attribute its existence to the lowest order of animals, and to plants; although, at the same time, they confess, that in these instances it is not attended with any of its proper phenomena. This is a mode of argument which does not admit of discussion; for, if we were to grant any premises that a person may please to assume, in contradiction to the evidence of our senses, our opponents might prove black white and white black, or any thing, however impossible. Besides, if we were sufficiently complacent to allow that inflammation could exist without exhibiting any of its sensible effects, nothing would be proved in favour of inflammation attended *with sensible effects*. An invisible or polypine inflammation, which admits of the animal being cut to peices, and turned inside out with impunity, would answer our purpose quite as well as none at all.

3dly. It has been conceded by some, that inflammation is *essentially* a disease, while, at the same time, they contend that it conduces to the healthy action of repairing injuries. Mr. Hunter gave origin to this error, by confounding adhesion and inflammation: yet he insisted on the impossibility of two different *diseased* actions, existing together in the same part, and preserving their distinctive characters. We

* See Medical Examiner, vol. 1.

can readily admit the addition of common inflammation to a specific disease aggravating and altering the natural course of the latter, or two diseased actions modifying each other so as to produce a third, not perfectly resembling either parent; but I cannot, by the utmost stretch of imagination, conceive the possibility of any species of disease producing healthy actions, except in the manner of derivation; or that health and disease can ever co-exist, without interfering, or being in the relation of opponents the one to the other. As well might adverse winds and tide serve to propel a ship; or a watch, with all its internal mechanism deranged, most accurately mark the time.

4thly. I have heard it said, that other surgeons hold the same opinions that I do respecting the treatment of injured parts; that, after all, it will be found that we agree as to the facts, and only employ different words to express our meaning.

If this had been true, we should long since have had lacerated wounds cured, without pain, protracted suppuration, and erysipelatous inflammation; and punctured wounds, without repeated abscesses and incisions, long suffering, sleepless nights, and sympathetic fever; which are well known to be the frequent consequences of such injuries, when treated by the advocates of inflammation. All persons who have experienced the healing of an open wound, on the anti-inflammatory plan, are well assured that there is something more than a difference of words between the two theories, and the modes of treatment necessarily resulting from them.

5thly. As coagulable lymph is sometimes effused during inflammation, and generally as a consequence of injury, and as it is always designed to form the means of uniting divided parts, it might seem, at a first view, that this effusion is the product of inflammation; and, at the same time, is necessary to conservation and cure. Such has been the confident conclusion of those who hold the opinion of inflammation being necessary to the healing of wounds. The co-existence or coincidence of two phenomena by no means, however, proves them to stand in the relation of cause and effect to each other. Neither does the precedence, as to time, of one event to another, show that the first is the cause of the second. All the circumstances must be taken into the account, before a correct judgment can be formed on this or any other subject. The conditions under which we observe the effusion of lymph to take place are three. 1st. When there is no appearance of inflammation whatever, as in the formation of the decidua uteri; 2d, previous to the time that inflammation could arise, as immediately on the receipt of extensive incised wounds more particularly; 3d, when inflammation has taken place, lymph is poured out to consolidate the cellular and serous tissues, and thus create a barrier, which will limit the inflammatory processes, or stop them altogether. Now, let us admit for a moment, that inflammation is the cause, and the effusion of lymph is one of its effects; in the first case I have mentioned, the effect would occur without the existence of the supposed cause; in the second, the effect would precede or prevent the cause; and in the third case, the effect would limit or arrest the progress of the cause.

Again, the cure of hydrocele has been exultingly quoted as the decided proof of the effusion of lymph being the product of inflammation, whereas, if we examine the matter fairly, the very opposite conclusion must be adopted; for example, in the cure by injection, the coagulable lymph serves to agglutinate the surfaces of the sac, and thus limits or prevents the inflammation, which would otherwise take place; and when the operation by incision, caustic, or the seton is employed, the cavity of the tunica vaginalis is obliterated by lymph being converted into granulations; and when the inflammation has subsided, the contracting, absorbing, and healing processes complete the cure. It is therefore found, that lymph is always thrown out for the purposes of reparation; and when it fails in accomplishing this, it is because it is opposed by inflammation. It is evident that the great distinction between all the other effusions, which are the real effects of inflammation, and that of lymph, is, that the former are constantly injurious, and never contribute to the cure. It forms no objection that a perfect cure cannot, under all the circumstances, be accomplished by the medium of lymph; for instance, when broken bones are so much displaced as to leave the limb deformed and almost useless, more lymph than usual is shed, and the processes of reparation are carried on with greater energy than if the ends of the bones were retained in exact contact.

I need not longer dwell on theoretic arguments. The important question, after all, whatever may be said on either side of the controversy, is simply this,—*can wounds of different kinds be cured in the human subject without inflammation?* No person, so far as I have learnt, has yet pretended to affirm the contrary, although some, who have never tried, have taken upon them to express doubts. I shall, however, go further, and assert that there never was an instance of any wound being perfectly repaired or healed until inflammation had subsided, or in which it did not exist, and I challenge the whole profession to produce a single example of the kind.

I now take my leave of the subject, being quite satisfied, if my book had never issued from the press, that a change to a mild plan of surgical treatment would eventually arise, from more enlarged views of living nature, and as a part of the general improvement already made in the management of the human kind under all other circumstances. The custom of promoting the inflammation of wounds, before they were allowed to heal, has descended to us from those dark ages of the world in which insanity was treated by the whip and chains, when people were forced to profess their belief of impossibilities by the rack or the faggot, when the of punishment death was awarded to almost every crime, when wars were accompanied with the cold-blooded butchery of women and infants, and when the education of the young was conducted by the ferula and the rod. All these thing are fast passing away. The humane spirit of the present age, and the universal spread of real knowledge, cannot fail to have their influence on the practice of surgery; and, at no very distant period, celebrity will be sought and obtained, not by dexterity in tormenting and carving the human body, but by scientific skill in the art of healing.—*London Lancet.*

---

*Treatment of Varicose Veins by Cauterisation with the Vienna Paste.*—The frequent occurrence of varicose veins in the lower extremities, affecting principally those persons who, from necessity, are compelled to undergo great fatigue, renders the possibility of radically curing them a subject worthy of serious consideration. The disadvantages, or rather dangers, attributed to the diverse surgical operations generally employed, have appeared to some surgeons sufficient to counterbalance any amelioration that might result from them, and to authorise their total rejection, substituting in their place a constant and moderate pressure. That compression, methodically applied, so as to give support to the limb generally, can alleviate, to a certain degree, the existing infirmity, is not to be denied; but, unfortunately, even this is of little avail under certain states of varix, as when, from some adventitious circumstance, or as a consequence of inflammation, a solution of continuity has taken place, giving rise to frequent, and sometimes alarming hæmorrhage, or more generally terminating in an ulcerous state, difficult in most cases to cure, and in some impossible, at least permanently, unless, as is a well-known fact, the varicose state of the surrounding veins, which is the principal cause of the ulcers not healing, be removed. To say that varix does not in itself constitute a disease, but is simply an infirmity, seems to us to be at best a play upon words, for, unfortunately, the difference between infirmity and disease cannot always be distinctly drawn; and in this case, that which would be simply an infirmity to a person above the necessity of exertion, becomes a disease, and a most serious one, to the individual compelled to daily labour; moreover, to assume as an invariable rule that, because, in some unfortunate case, death has resulted from our endeavours, we are on that account not justified in attempting to cure varicose veins, appears to us to be an unsound maxim, unworthy of the surgical art, as will forcibly strike any one who has had opportunities of witnessing the miserable state of persons thus affected, and who cry out for some relief, not to their sufferings, but to the infirmity, or, rather, disease, which precludes them from labour.

Amongst the various remedies that have been successfully employed, cauterisation of the varix with the caustic potash, seems to us to have been cast into an undeserved oblivion. First employed by Ambrose Paré and Guillemeau, it remained unnoticed until Sir B. Brodie re-examined its efficacy; but he mentions it only to blame, for according to him the cure of the varix affords but an inadequate compensation for the pain produced by the caustic, and the inconvenience arising from the tedious healing of the ulcer, which remains after the slough. More recently, Mr. Bonnet, surgeon of the hospital of Lyons, made several experiments, the result of which he published in the "Archives Gen. de Med." of May and June, 1839; but it is not to the merits of this plan that we wish to call the attention of the medical world, but to a modification which to us appears important, recently introduced by Mr. Laugier, surgeon of the Hôp. Beaujon at Paris.

As we have already stated, the objections made by Sir B. Brodie to the use of the caustic potash are: 1st, the pain; and, secondly, the length of time required for the healing of the ulcer resulting from the slough. Mr. Laugier imagined that if, before applying the caustic, the skin should be divided down to the vein, a smaller quantity of caustic would be required, its action more prompt, and the slough necessarily smaller. He also substituted a composition, consisting of equal parts of quick lime and caustic potash, to the caustic potash alone; the advantages he attributes to their composition, known under the name of Vienna paste, are, 1st, a more rapid and certain action; the caustic potash, when employed alone, as was remarked by Mr. Bonnet, sometimes did not cauterise sufficiently deep, and required two or three applications; and, 2ndly, the production of less pain.

Several other objections can also be made against the use of the potassa fusa, according to the old plan. From the extent and depth of the sloughs, Mr. Bonnet derived two laws: 1st, that the caustic can only be applied on veins corresponding to muscles, from fear of affecting the periosteum; 2ndly, that, except in cases of spontaneous hæmorrhage, the internal saphena should not be cauterised whenever the external also was affected with varices, since the latter, the circulation in the internal saphena, being interrupted, could but increase. Mr. Laugier's plan obviates these inconveniences completely; the action of the paste being prompt and easily localized, the quantity employed being small, the sloughs being necessarily limited, and the loss of skin almost null, we are enabled to operate not only in front of the tibia and of the condyle of the femur, but also on both saphenæ at the same time, without fear of affecting the bone, or of depriving the limb of too great an extent of skin.

But the most essential advantage in the method of Mr. Laugier is, that by the immediate and prompt cauterisation of the vein, the danger of severe phlebitis is avoided. In applying the caustic potash according to the ancient method, it often happens that its action does not extend sufficiently, and that the vein is either opened or simply laid bare; circumstances, it is evident, tending to produce diffuse inflammation much more than if the vein had been totally disorganised. By the immediate application of the paste, the vein is deeply cauterised, and in twenty minutes, or at most half an hour, a coagulum has formed, which, according to Mr. Laugier, has the advantage of isolating before inflammation of its walls has supervened, the disorganized portion of vein from the general venous system and the circulating fluid thus preventing the absorption of pus, if any should form, into the system; in fact, circumscribing the inflammatory process, and preventing its becoming diffuse.

Another important consideration is the possibility of achieving a radical cure. The effect of every mechanical injury to a venous trunk is the production of inflammation of its lining membrane, more or less severe, known under the term phlebitis; but putting aside the severe form of inflammation, and confining ourselves to the consideration of what is called adhesive inflammation, it will be seen that the first appearance is the deposition of a certain quantity of coagulable lymph, extending more or less along the cavity of the vein, according to the extent of the adhesive inflammation itself, necessarily producing agglutination of the opposite walls of the vein, and, if uninterrupted, obliteration of the venous canal. But as, in the union of wounds, circumstances may arise to disturb their beneficial march, and the agglutination of the venous walls may not be sufficient to resist the impulse of the column of blood when exertion is made, or the obliteration of the canal may simply be partial, extending only for a few lines, the remainder of the canal remaining pervious, and still giving passage to the blood when brought by the neighbouring anastomosing branches. This, as daily experience shows, often happens after treatment of varices by the ordinary methods, especially after the use of pins, or after simple division of the vein; but, from the observations of Messrs. Bonnet and Laugier, it would seem that cauterisation, when effectually made, disorganises completely the vein, and determines a total obliteration of its canal through the whole extent comprised between the cauterised spots, and in neighbouring portions of vein, or, at least, to such an extent, above and below the eschars, as to render a return of the disease in the same veins almost, if not totally, impossible.

The following is the plan that Mr. Laugier adopts in operating:—A fold of skin, either transverse or in the direction of the vein, being raised, a small incision from five to six lines is made, and the vein laid bare. The slight hæmorrhage that results from the incision is immediately arrested by the nitrate of silver; for it was remarked by Mr. L., that the blood diluted the paste, and impaired considerably its agency. The lips of the wound are then covered with small slips of charpie, to defend them from the action of the caustic, and the wound is filled with the paste, generally requiring about the size of a small hazel-nut: the action of the caustic creates but little pain, and, in fact, most of the patients find the application of the nitrate of silver the most painful part of the operation.

The number of applications must depend on the extent of the varices, but generally on the first day only two are made on the most prominent varix, about six or seven inches distant from each other; the subsequent applications must depend on the state of the limb. It is requisite that the patient should remain perfectly quiet, until the caustic has formed an eschar, to prevent the paste from escaping, and destroying the neighbouring skin: this requires from thirty to forty minutes, and then the eschars, being cleaned of the remaining paste, are covered with diachylum. It is, of course, necessary that the patient should remain in bed until the sloughs are fallen; he then is allowed to make moderate exertion, and, when no untoward circumstances arise, the cure is generally effected in six weeks or two months.

In a future communication some cases will be forwarded: already from thirty to forty patients have been operated on by Mr. L., and no accidents have as yet occurred; nevertheless, two deaths have taken place during the treatment, but under circumstances entirely unconnected with the operation. We will mention them in our next, as they are interesting, inasmuch as they show the state of the venous canal, after the application of caustic. Most of the patients operated on have been perfectly cured; and in those cases in which the varices have not been totally obliterated, such an amelioration has been effected, as to render the patients capable of returning to laborious occupations, which previously they were forced to desist from.—*Ib.*

---

*Morbus Coxæ successfully treated with Mercury.* By O'Bryen Bellingham, M. D., one of the Surgeons of the Hospital.—The following case of disease of the hip-joint, arrested in its early stage, by the exhibition of mercury, upon the plan first introduced and successfully put in practice by Dr. O'Beirne, of this city, possesses some features of novelty, and presents some points of difference from those which have been already published.

In the first place, it goes far to prove that mercury, carried to the extent of salivation, with rest in the horizontal posture, is quite adequate to effect the cure of morbus coxæ in its first stage, without local bleeding in any form, or counter-irritation in any shape. The patient, previous to the present attack, had laboured under hip-joint disease upon the opposite side, which had gone through its several stages, and had ended in permanent shortening and deformity of the limb. But few cases of cure are, I believe, upon record.

James Tight, aged 10, a scrofulous-looking boy, with red hair and blue eyes, admitted into St. Vincent's Hospital, March 19, 1840. His mother states that six years ago he laboured under hip-disease upon the left side, which ended in a shortening of the limb to the extent of three inches; the leg and thigh are, in addition, wasted, and the thigh is permanently semi-flexed. Within the last six weeks, he has suffered from weakness of the right limb; a fortnight ago, his crutch slipped from under him, and he fell upon the ground. Since then he has complained much of pain in the right hip, which is more severe at night than in the day, and he cannot bear his weight upon the limb. On one day only he says he suffered from pain in the knee. He complains of pain when the articular cartilages are pressed against each other, or when pressure is made upon the great trochanter, or in the groin. He has lost flesh and appetite, the buttock is flattened; but no comparison can be made with the opposite side, on account of the condition of the limb. His mother says, that for the last fortnight he has moaned constantly during the night, and that the symptoms are similar to those with which the disease set in upon the opposite side.

He was directed to remain in bed, and take a pill containing calomel, gr. ij., and opium half a grain, night and morning.

March 20. Slept better last night than he had for the last fortnight.

21. Bowels moved once by the medicine; had no pain last night.

23. He told me that he got up yesterday, and was able to walk with the assistance of his crutch. He suffers no pain now when pressure is made in the groin, or when the head of the femur is pressed pretty firmly against the acetabulum.

25. His mouth is sore; says he can walk as well as ever; has no pain at all at night, and rests well. His mouth to be gargled with a solution of alum, and let him take but one pill in the day.

26. Mouth better. To take a pill twice a-day.

28. Bears strong pressure of the head of femur against the acetabulum; also strong pressure over the trochanter major, and in the groin, without complaining.

April 2. Says he is quite well; mouth sorer. Discontinue the pills.

6. Says he can walk now without his crutch; suffers no pain of any kind; mouth nearly well; appetite good. To remain still in bed.

9. No pain felt on any motion of the joint; a small gland in the groin has enlarged and become painful, within the last day or two. Let two leeches be applied to the right groin.

11. The surface, for about a hand-breadth around the leech-bites, has an erysipelatous blush, and is painful to the touch. Let him take a tea-spoonful of the following mixture every hour:

Tartarised antimony, grs. ij;
Distilled water, ℥iv.

14. The erysipelas has altogether disappeared. Discontinue the solution of tartarised antimony.

18. He has remained in bed since; is quite well, and is able to go about without his crutch.

Dismissed, with the recommendation not to exercise the limb much for at least another fortnight.—*Ib.*

---

*Monæsia.*—At the last meeting of the Royal Medico-Botanical Society, Dr. Sigmund laid upon the table several pharmaceutical preparations, which had been sent for the examination of the members by M. Derosne, who had obtained them from a new drug recently imported into France from South America, under the name of monæsia. Dr. Sigmond observed, that a specimen of the extract had been some time time since submitted to his inspection, by a gentleman who had received it from Dublin, and he must confess that the opinion he had then formed of it was, that it was a compound substance, and not the product of any one vegetable. This idea was founded upon the external appearance, and upon the taste of the drug, not upon any analysis, or minute examination of its structure, for the quantity shown to him was very small. He was persuaded, however, that from the quarter from which this new medicine emanated, there could be no attempt to deceive the medical profession; for it was to an individual whose family were well known as pharmaceutical inquirers, that they were indebted for the present discovery. From the nephew of Derosne, whose labours upon opium, and whose analysis had led the way to our present knowledge of morphia and narcotine, we could only expect truth. The four preparations now placed before them were an aqueous extract, a hydro-alcoholic tincture, an ointment containing an eighth of its weight of extract, and an acrid principle which is obtained by analysis. But Dr. Sigmond could not but express his regret that no specimen of the bark from which monæsia is supposed to be obtained, was placed before them; M. Derosne had, however, promised that at a very early period he would supply this deficiency; and as that gentleman was present at the meeting, he would, although he was unable to address them in English, give them every satisfactory information upon this point. It would appear, from a monograph now placed before the Society, that a French m rchant, residing in South America, having witnessed the good effects of a substance employed by the natives in cases of dysentery and of diarrhœa, sent a quantity of the drug to Paris, and it was placed in the hands of M. Derosne, who found it to be an astringent endowed with a sweet taste. About a year and a half after this, he obtained some specimens of the bark of the tree, from which he procured an extract, presenting all the characters of the extract previously sent to him, and likewise identical in its composition.

M. Derosne had stated that the pieces of bark were of a deep red colour, of a smooth fracture, and that they were evidently the produce of a tree of considerable dimension, and probably belonging to the mimosæ. Chemical analysis had shown the bark to consist of chlorophylle, of vegetable wax, of a fatty crystallisable matter, of glycyrrhizine, of an acrid matter somewhat bitter, of a little tannin, of a natural acid not yet examined, of phosphates of lime and magnesia, of a colouring matter analogous to that of cinchona, and, lastly, of salts of lime in combination with the natural acid. In France the preparations had been carefully tried by numerous physicians, amongst them Baron, Lisfranc, Manec, Laurand, and Payen, and they were led to form a high opinion of their effects, more especially in various affections of the digestive organs; it was serviceable in bronchitis, in phthisis, in hæmoptysis, and as an astringent generally. Its external application, in various affections of the skin, had been attended with success. It would appear, that although exerting considerable influence upon the system, it had never produced any bad symptom. The doses were, of the extract, given in the form of pills, from six to twenty grains; as much as forty-five grains had been given in a day; of the tincture, from a drachm to a drachm and a half, in six ounces of water. Dr. Sigmond referred to the experiments that had been made in Dublin, from which it appeared that much benefit had been derived from it as an astringent. M. Derosne would gladly furnish any member with a supply, who would make public the result of his experiments. He concluded by again expressing his hope, that the bark would be furnished to them at an early period; for, without that was laid before them, he felt persuaded that the Society could not with propriety investigate the subject further, although its members would gladly try the effects of any medicine which brought with it such recommendations from their medical brethren in France.—*Ib.*

---

*Deafness produced by Quinine.*—In a recent number of the Lancet is related a case of dumbness, caused by large doses of quinine. I have lately had my attention directed to two cases of deafness, induced by large and repeated doses of that medicine.

The first patient, a female, æt. 48, had been suffering, for some time, from sciatica, for which quinine was administered. After taking it for about three weeks, deafness suddenly ensued. It was thought proper to subject her to no remedial treatment, with the exception of an occasional aperient. In about three weeks she regained her hearing.

The second patient, also a female, æt. 60, had been suffering considerably from ticdouloreux, and quinine had been taken freely and frequently; in consequence, the acoustic nerve gradually became more and more *insensible*, until conversation was entirely prevented. Unfortunately, this lady continued the quinine for a few days after the deafness had commenced. The quinine was then withdrawn, but she was subjected to no other remedial treatment. In about three weeks her hearing began gradually to return, and we were in hopes that she would entirely regain it; this, however, has not been the case, she being only capable of hearing those persons who address her in a very loud tone.

These cases are doubly instructive, as they suggest to us the propriety of not only withdrawing, in similar cases, a medicine which possesses so great a power over the special nerves of sense, but we may naturally expect that, in many cases of tinnitus, its free and frequent exhibition may remove this unceasingly tormenting symptom, when dependent upon functional derangement of the acoustic nerve.

The latter case, more particularly, suggests the importance of not alone trusting to nature to restore the defective energy of the acoustic nerve, when injured by the action of quinine. In addition to aperients, which should be administered for several alternate days, leeches should be applied over the mastoid processes, or, if occurring in a young and healthy subject, cupping-glasses might be placed behind the ears, afterwards applying strychnia in the endermic method. If, after a few weeks, the hearing should only partially return, it would be a very proper case for the application of electricity, in the manner recommended by Magendie.—*Ibid.*

---

*On the Treatment of some Diseases of the Testicle by Compression.* By Langston Parker, Esq., Surgeon, of Birmingham.—A short time since, Fricke, of Hamburg, proposed the treatment of hernia humoralis, by compression; and, subsequently, Ricord has modified Fricke's method, which is now generally followed with success in the Parisian Civil Venereal Hospital.

I have now for some time adopted this practice, and can speak from my own experience, in numerous instances, to the advantages it possesses over the ordinary methods, both as regards the number of complete cures effected, and the rapidity with which they are made. Fricke originally proposed compression in ordinary swelled testicle; but I have since employed it in chronic or subacute inflammations of this organ, whether dependent on syphilitic causes or not.

In common swelled testicle compression may be employed as soon as the patient is able to bear the application of the plasters; and this is generally much sooner than might be supposed, however acute the disease may be. The great advantages possessed by compression are, almost immediate relief to pain; which is occasioned, in great measure, by the dragging and weight of the inflamed organ, and is mitigated, and most commonly completely removed by its support: the patient can pursue, in most instances, his ordinary duties, and there is no occasion to confine him to bed; a circumstance of great importance where concealment is wished, as it always is, in diseases of this kind. In addition, the cure is much more quickly, and much more perfectly effected by compression than under the ordinary method, the swelling of the gland more rapidly subsiding, and less commonly leaving behind it any chronic irritation and enlargement, which is liable at some future time, in bad constitutions, from injury, or from a renewal of the disease, to degenerate into some of the malignant forms of disease of the testis.

Chronic inflammation and enlargement of the testis also very commonly occurs as one of the symptoms of constitutional syphilis, as well as from other causes. Here, also, compression, as a local remedy, is by far the most valuable of any with which I am acquainted, particularly in those cases where enlargement of the organ, from chronic inflammation, has taken place to a considerable extent, where the weight chiefly incommodes the patient, and little or no tenderness is experienced when the part is handled.

I am not prepared to say how far compression may be of service in the incipient stages of certain forms of malignant disease of the testis, but believe much advantage may be derived from its employment, since, in addition to the benefit to be looked for from the mere compression of the part, much good may be expected from the constant topical application of mercury, iodine, and other remedies, only to be well accomplished in this way.

Compression of the testis is to be practised by surrounding the organ by straps of plaster, applied as closely and as tight as the patient can bear. The plasters I generally use are those of ammoniacum, with mercury, or iodine and belladonna. These are to be smoothly spread upon thick wash leather, and cut into thin straps; an assistant then grasps the testicle, and draws it as far downwards as he can in the scrotum, which should previously be washed with a little spirits of wine. The first strap should be placed at the upper part, circularly round the testis, and succeeded by others, placed in the same direction, till the whole is covered. A second series of straps is then to be placed in an opposite direction to the former, crossing them at right angles, from behind forwards. One or two long ones may then be placed over these, where they appear to be most needed, to keep the whole in place. The parts should be supported by a well-fitting suspensory bandage, although the plasters at once relieve any inconvenience or pain that may have been occasioned by the dragging or weight of the testicle.—*Ibid.*

---

*Gangrene of the Mouth successfully treated by the the Actual Cautery.* By Henry Obre, Esq., House Surgeon to the Marylebone Infirmary.—March 18. Charles Harris, aged nine years, living in a damp, unhealthy atmosphere, was attacked a few days since with fever, which quickly terminated in typhus. He was in a low insensible state for two or three days, requiring wine and stimulants to recover him. When sufficiently recovered to sit up in bed, on the 25th, an ulceration was perceived on the external and back part of the gum of the left upper maxilla, having an ash colour. This had increased to such an extent, before being discovered, that the two first molar teeth were loose, and soon fell out; the fauces were covered with apthæ, the tongue and other parts of the mouth remaining perfectly natural. The ulcerated surface was touched with muriatic acid, mixed with honey; and he continued the use of quinine and red wine, which he has been taking since.

26. The ulceration has passed to the mucous membrane of the cheek, which is hard, swollen, and has a glazed appearance externally, except in one point in the centre, which feels soft, as if the disease had nearly passed through; pulse small; no diarrhœa, perspiration, or any symptom of debility. Nitric acid was applied to the ulcerated surface, the mouth cleansed with a gargle of bark and alum, and a blister applied to the back of the neck.

29. The gangrene has passed through the cheek to day; its size is about that of a half-crown; it presents a dark colour, and gangreneous fœtor; the hands require to be restrained to prevent his disturbing a poultice of yeast which has been applied; the ulcer to be washed with a solution of chloride of soda; he sits up in bed, and eats mutton, chops, indeed any thing he can get, his appetite being seldom satisfied.

30. The disease has increased in extent, nearly joining the commissure of the mouth in front, and passing back to within an inch of the tragus. The parents having consented, the actual cautery was applied to the diseased surface, on the external parts, with little uneasiness to the child. From this time, until the 4th of April, the disease was perfectly arrested, when it began to increase under the integument. The edges of the sore were irregular and everted, the internal parts of the mouth quite exposed from the wound, and also the superior maxilla as high as the zygoma. Being afraid that the lower eyelid would be destroyed, as the disease was extending in that direction, the cautery was repeated with the same success as previously. Charcoal was ordered to be sprinkled on the poultice; the smell of the chlorine being unpleasant.

8. The greater part of the slough produced by the last application is coming away; granulations on many parts are making their appearance; perspirations have come on this last day or two; in other respects he is improving, eats, sleeps and drinks well. Another of the double teeth on the diseased side has fallen out.

Those parts not showing a disposition to heal, on the 10th were touched with nitromuriatic acid. The perspirations have left him; had an attack of diarrhœa this morning, for which he was ordered aromatic confection. The boy is up and walking about the room; continues the use of wine, meat, &c. From this time the wound gradually improved, and he attended at the infirmary two or three times a week; the face being perfectly healed, excepting a small part the size of a pea. He is unable to open the mouth to a greater extent than half an inch, in consequence of an adhesion of the cheek near the mouth to the gums on the diseased side, which can easily be removed by the division of the adhesion. The face is not disfigured to the extent that would have been supposed.

On the 2d of January, 1840, Margaret Dagnell, aged three years, became my patient, when the following history was elicited from her parents, who are labouring people: Previous to the present illness, the child was never seriously indisposed, always resided in a confined neighbourhood, either in London or Manchester, her diet being chiefly vegetable; she seldom took animal food, in consequence of their poverty. About a month since, an epidemic of measles prevailing, she was attacked slightly, being ill only a few days. While convalescent, some small spots or ulcerations were observed on the inner surface of the lower lip and gums, for which the child was taken to a druggist, who ordered a bitter mixture; no other medicine being given. The ulceration now rapidly increased, and destroyed part of the lower lips and gum, loosening the lower teeth, all of which fell out in a few days, except the first molar on each side.

The child lays on the back, appearing to suffer little or no pain; the lower part of the face has a frightful aspect, the covering of the lower jaw anterior to the insertion of the masseter is quite denuded, except a narrow communication joining the angles of the mouth; the exposed bone beginning to decay; the integument surrounding the disease is pale, tumid, and hardened; fœtor most unpleasant; pulse, 108; tongue covered with a brown fur; the hands are continually thrown about, and attempts made to pick her face; wine and quinine were given internally; warm applications to the face.

3. The gangrene much increased, has destroyed the communication between the angles of the mouth, and separated the base of the tongue from the bone, passing down nearly to the os hyoides, exposing the sub-maxillary gland, which is but little affected. The actual cautery was applied all over the diseased surface on the external parts, which seemed not to be perceived by the child; chlorine cloths were applied, and the mouth washed with a gargle of bark. A quantity of sanious fluid flowed through the wound.

4. The gangrene has not extended externally, where the cautery has been applied; not so inside the mouth, which has been touched with nitric acid to-day; the child is fed with fluids, the head being held back to prevent their flowing out through the chasm. The child remained in this state until the 6th, when it died, exhausted by diarrhœa; the toes of the left foot, for a day or two before death, were cold and blue; flannel was kept applied to them; the teeth and gums of the upper jaw remain perfect.

*Remarks.*—The cases which I have related above, demonstrate the very great efficacy of the actual cautery in extensive destruction of the face from gangrena oris. I was induced to employ the actual cautery on the recommendation of Dr. P. Hennis Green, who informed me that he considered it to be the only remedy which afforded any chance of arresting the gangrenous destruction of the soft parts, after perforation of the cheek. In the first case a complete cure was obtained; in the second the child died, but the cautery completely arrested mortification at the parts to which it was applied, and would, probably, have been attended with the same success as in the first case, if it had been employed under equally favourable circumstances. I am not aware that the actual cautery has ever been used in this country in the treatment of gangrena oris, although most of our writers recommend it, on the testimony of Baron, Isnard, and Marjolin. It is evidently a powerful agent, and is worthy of trial in that severe form of a disease, which, according to Dr. Willis, destroys nineteen out of twenty of those attacked.—*Ibid.*

*Luxation of the Odontoid Process; Pressure on the Spinal Marrow, without Symptoms.*—The political jonrnals have alluded to a very remarkable case of luxation of the axis, with considerable displacement of the odontoid process, which by its pressure had reduced the spinal marrow to nearly one-third of its bulk; yet, although the accident dated from several years, no symptoms of paralysis had occurred during life. This case has been the subject of medico-legal investigation before the medical coroner for Middlesex. A very similar one is related in the April number of the "Edinburgh Journal." A young man, 22 years of age, was confined to bed for seven weeks by rheumatism. On becoming convalescent, it was discovered that anchylosis of the superior vertebræ had taken place, the head being fixed and bent forwards. The man was able, however, to return to his occupation of cutler; at the age of 28 he was seized with severe bronchitis, which recurred occasionally for four years, when he died suddenly in a fit of asphyxia. On examining the body after death, besides extensive anchylosis of the upper cervical vertebræ, there was luxation of the axis; the odontoid process was thrown backwards, so as to approach within three lines of the anterior surface of the posterior segment of the atlas. Notwithstanding this remarkable diminution of diameter, the membranes and substance of the spinal marrow, at this point, appeared to be perfectly healthy, and the nerves arising from the cervical portion of the cord, presented their normal size, colour, and consistency.

*Ibid.*

---

*Puncture for Chronic Hydrocephalus.*—In the same number of the "Edinburgh Journal," are recorded the histories of two cases of chronic hydrocephalus, treated by puncture. The subject of the first case was two months old; the head measured 20 inches round, and 12 inches from ear to ear. On the 10th of March, 1839, a puncture was made, with a trochar, on the right side of the head, and 15 ounces of clear fluid were drawn off; the child turned pale and vomited. The head was now bandaged up. On the 23d, 11 ounces of fluid were evacuated; on the 26th, 16 ounces. The child was again visited on the 21st of August, and then appeared to be in good health, although the head had increased in volume to 27 inches by 17. Since then the medical attendant lost sight of the case.

In the second case, the child, 8 months old, had been attacked 10 weeks after birth; the head measured 22 inches round, and 14 from ear to ear. On the 12th of April, 1838, 20 ounces of fluid were drawn off by puncture, and the head strongly bandaged; on the 28th, 23 ounces; on the 12th May, 22 ounces; on the 19th, 19 ounces. These repeated punctures and extraction of fluid were attended with very little benefit. On the 29th, the head being again measured, it was found that only half an inch had been gained. Mercury was administered, but the child sunk on the 2nd of June. On examining the brain after death, 90 ounces of serum were discovered in the cranial cavity.—*Archiv. Gen. de Med.*, May, 1840.

---

*Preservative Powers of the Vaccine Virus.*—The question of the preservative efficacy of the vaccine virus aganst small-pox, has been frequently discussed of late, and much difference of opinion expressed by various medical authorities. In the last number (June 13, 1840,) of the French "Medical Gazette," now before us, we find some remarks on an epidemic of small-pox, which prevailed for nine months in one of the French provincial towns; these refer principally to the protective influence of vaccination, and are based, as the writer assures us, on considerable experience. His conclusions are the following:—

1. The epidemic prevailed amongst the vaccinated and the non-vaccinated, but was most fatal amonst the latter class.

2. Amongst the non-vaccinated numerous cases of confluent small-pox occurred; but their fatal termination depended rather on the supervention of catarrhal symptoms than on accidents connected with the small-pox itself.

3. The number of vaccinated persons attacked by variola was very great, chiefly from the age of eight or nine years, up to that of thirty. But the more removed were the patients from the period of vaccination, the more severe and dangerous was the attack of small-pox.

4. Again, the lesser the period since vaccination, the more mild was the disease, and the more rapidly did it terminate, never extending beyond nine to eleven days.

From these and several other facts of a sinilar nature, the writer concludes that the preservative power of the vaccine virus has considerably diminished within the last forty years. In support of this opinion, he described the appearances of the vaccine vesicle such as he was accustomed to see it twenty years ago, and compares them with the progress of the same vesicle in our own more immediate time. Formerly the local and general symptoms were very well marked and energetic, now they are of an insignificant character; hence the author insists on the necessity of renewing the vaccine matter from its original source.—*Gaz. Med.*, No. 24, 1840.

---

*Anatomy of the Medulla Oblongata.*—At the Surgical Society of Ireland, May 9, 1840, Dr. Power said he would exhibit a very remarkable preparation which he had accidentally discovered in the Museum of the Richmond Hospital. On examining the preparation, he was struck with the appearance presented by the medulla oblongata. The fact he was about to mention was alluded to by Mr. Solly, who deserved the credit of having first particularly pointed it out. In the one hundred and fifty-fifth page of Mr. Solly's work, speaking of the anterior columns, or motory tracts of the spinal cord, he makes the following observations:—"Of the fibres, which run from the antero-lateral columns to the cerebellum, there are evidently two sets, one superficial and one deep. The superficial may again be divided into two sets; the first cross the surface of the cord, *immediately below the corpus olivare, and may generally be seen without dissection;* they are more distinct in the sheep; bullock, and horse, than in man, in whom they form a very thin layer, emanating from the corpora pyramidalia; and I have no doubt, that they actually decussate with their fellows of the opposite side, forming, in fact, part of the apparatus of decussation, though I have not yet postively ascertained the fact. In his sixth plate (fig. 1) he has given a representation of these superficial fibres, which pass from the copora pyramidalia, to become continued with the productions of the corpora restiformia into the cerebellum. The preparation exhibited by Dr. Power, showed these fibres of communicaton in a most satisfactory manner, and much more distinctly than did Mr. Solly's plate. In this preparation the corpus pyramidale of the left side was seen to consist of two distinct bundles of fibres, an anterior or superficial, and a posterior or deep set. The superficial was perfectly distinct from the deep set, and formed a well-marked flattened band about a quarter of an inch in breadth, which first ran downwards, and then turning underneath the corpus olivare, became continuous with the corpus restiforme; thus describing an arch, the concavity of which embraced the inferior extremity of the corpus olivare; the deep set followed on its direct course through the pons Varolii. Thus the usual prominent appearance of the corpus pyramidale on that side seemed to be altogether lost. These points were exceedingly well marked in the preparation. Mr. Solly states, that the arrangement is a common one, and always discoverable, even without dissection.—*Reported in Dublin Medical Press*, July 1.

---

*Mr. Phillips on Wounds of Joints.*—Wounds of joints may be contused, incised, or lacerated; may be penetrating or non-penetrating.

So long as the wound does not directly implicate the tissues of the joint, it should be treated much like a wound elsewhere; still, as tissues around a joint do not quickly take on inflammatory action, it often ends in suppuration when they do. The form and mobility of joints oppose difficulties to the proper application of bandages, and there is, therefore, greater probability of the lips of the wound not being kept in apposition.

*Penetrating* wounds are very serious injuries; ginglymoidal articulations being more superficial than orbicular, are more frequently the seat of these injuries. In many species of occupation, the ankle and knee joint are particularly exposed; among ship-carpenters, who place under their foot the piece of wood they are shaping with the adze, the ankle joint frequently suffers; among vine dressers the knee joint is often wounded. Whatever the joint which has suffered, the danger depends upon the intensity or kind of the inflammation which is developed. This inflammation is sometimes very violent, and reasons for this have not been wanting. The ancients, who were impressed with the great danger of wounding tendons and aponeuroses, were impressed with the idea that the injury to those tissues around a joint completely accounted for the occasional violence of the symptoms. The resistance offered by those tissues, and the violence necessary to overcome it, is another and probably a better reason. The action of atmospheric air upon the tissues of the joint was regarded by Munro, Thomson, Bell, and others, as the sole reasons for the symptoms. Others have attributed them to the action of air and the contact of the dressing, which, probably, is nearer the truth than any of the others; but it is a matter of question whether air alone can exercise a very deleterious influence upon articular or synovial surfaces in a healthy state. When we amputate at the tarsus, at the knee, the shoulder, or the hip-joint, we do not find such serious symptoms developed; but if we open a joint in which there is an accumulation of synovial or purulent fluid, and maintain the communication, a certain quantity of inflammatory action is certainly developed. The fluid is altered, it is in direct contact with the almost inert surfaces of the cartilage on the one hand, and on the other with the coverings of the joint, which inflame, and acute symptoms are manifested. Certain it is, we may open a joint many times to remove a loose cartilage, without exciting inflammatory action; it is equally certain that when a joint is distended by synovia or pus, we cannot open it with the same impunity. We may perforate the pleura of a healthy animal twenty times, and, probably, pleuritis will not be developed in one, but previously inflame the pleura, in a single case, and when pleuritis is developed, make a similar perforation, and the chances are suffocation will follow in a few hours. I believe, therefore, that the action of atmospheric air is to be feared only when the tissues are already inflamed, or when a fluid which has undergone change is found in the joint.

*Symptoms.*—The first symptoms of penetrating wounds are often not different from those of ordinary wounds; the wound not being extensive, and the patient being unable to appreciate the importance of the escape of synovia, he uses the limb in fatal security. During the first two or three days there is nothing to alarm; but pass this period, in three, four, or six days after, symptoms are manifested which show the gravity of the injury; there is itching about the wound, there is stiffness and some tumefaction, the edges are tumid, and a reddish serum escapes in a considerable quantity; these symptoms may yield, if arthritis be not developed; but if inflammation sets in, the pulse becomes strong, the skin hot, the face injected, the tongue white, with thirst, loss of appetite, and sleeplessness, if the joint be large; if it be small, the symptoms creep on in a more insidious manner, and gradually extend to contiguous structures, and often give rise to numerous abscesses, before attention has been directed to the mischief. Besides these consequences, contused or lacerated wounds of small joints are occasionally followed by tetanic symptoms; this is the general experience of military surgeons. In the larger joints, when the symptoms of arthritis are set up, the patient keeps his bed, his limb is semi-flexed, the joint tumid, the colour of the skin covering it is often unchanged, though tense and glistening; the pain becomes more acute, increasing in proportion to the intensity of the inflammation, becoming more aggravated as it extends to the ligamentary and fibro-cartilaginous tissues. Often it extends beyond the joint, especially upwards; abscesses are formed so insidiously that until fluctuation is apparent they are often hardly suspected. It is singular, in many cases these abscesses are developed about the middle of the limb, apparently without direct connection with the joint, unless in some cases where the distended capsule has given way. Usually these abscesses are formed in the subcutaneous tissues and muscular interstices, and then the pus burrows with great facility.

*Diagnosis.*—The diagnosis of these wounds is usually easy; their situation, the tissues divided, the fluid which escapes, and the appearance of the parts, are usually sufficient to lead us to a right conclusion. In punctured wounds difficulties will arise; it is true these difficulties may be removed by the introduction of a probe; but as a probe may cause serious injury, it should not be wantonly used. If the wound be large, the edges may be separated, so as to see the cartilages. The escape of synovia is an important circumstance, but, it cannot be considered as pathognomonic, because it may follow the wound of a tendinous sheath, the joint itself being uninjured, or it may be so little, or so mixed with other fluids, as not to be recognised; and, in a large wound, after twenty-four hours, inflammation would work a great change in its characters.

It is singular how long a tendency there seems to be, apparently from a kind of sympathy, when there is suppuration at one point, to repeat a similar action in others, or even in serous sacs; how this happens I cannot tell; I merely record the fact.

*Prognosis.*—Formerly all wounds of joints were regarded with great apprehension, and unquestionably in most cases they are very serious injuries, but less fatal than our predecessors seemed to think. John Bell and Ledran stated that an opening made into a joint in a state of suppuration was necessarily mortal. If, instead of saying so, they had contented themselves with stating that complete cure was improbable, they would have been nearer the truth; for there would be no difficulty in mentioning a great number of cases of every variety of wounds of joints which have done well; and unquestionably, in many cases of great distention of joints from purulent or other collections, instant relief may be obtained from a puncture or incision judiciously made. But we must inquire further; what we want to know is, when a joint, such as the knee, suppurates in consequence of a wound, what is likely to happen? I apprehend death almost inevitably, unless the limb be removed, and as a rule even in smaller joints.

The inflammation succeeding to a wound of a joint being the type of the *anatomical changes* produced by these from other causes, I shall here point them out. If the patient die soon after a joint is wounded, the only appreciable alteration is in the fluid contained in the joint; it is turbid, serous, and yellowish. If examined at a later period the internal synovial surface loses its polish; it is rough, and covered by a fibrino-albuminous coating; at a still later period, the synovial membrane may be thickened, the subjacent cellular tissue may be infiltrated, the contained fluid may be puriform, and, at a later period, purulent; the synovial membrane no longer retains the character of a serous tissue, but is more like a mucous surface streaked with blood; the cartilages may be thinned, softened, or destroyed; the ligaments lose their colour and consistency; the bones may be carious.

*Treatment.*—Inflammation of synovial surfaces being so serious, what should we do to prevent it? If the wound be simple, endeavour to heal it as quickly as possible, taking care that the position is easy, and the rest absolute. When the wound is brought together, some surgeons use bandages moistened with white of egg, to secure the exclusion of air. If the wound be irregular, contused, or lacerated, this may be impracticable, and we must then leave much to nature, arranging the position and apparatus so that the fluids which are secreted may find a ready exit. It may become necessary to moderate inflammatory action at the part; and for this purpose Schmucker thought there was no agent possessed of such efficacy as water. It is said that of sixty soldiers treated in this manner in 1792, at the convent of Cousarrebrouck, only four died, whilst in the French camp, of those similarly wounded, nearly all died. I have no doubt of its value, but I have already, more than once, stated my objections to it. The greater number of surgeons prefer local or general bleeding, with or without cold, with or without emeto-purgatives and rigid diet. Dorsey and Fleury liken synovial to pleural inflammation, and think repeated blistering upon the wound itself, or the point most acutely inflamed, the best mode of treatment. We have not had enough of experience of its results, in acute inflammation of joints, to speak confidently on the subject, but so far as that experience goes, it is a most valuable agent. In Rust's Magazine is a proposition by Schrager, which consists in applying upon the wound nitric acid "to prevent morbid secretion." He mentions two cases of cure, which I fancy might have got well without it. In cases where the inflammation is not arrested, the tumefaction being considerable, and the pain great, it may be necessary to make warm emollient or narcotic applications to the part. If suppuration supervene, the communication with the exterior not being such as to allow of the escape of the purulent fluid, the fluctuation being evident and the distension distressing, it is proper to make an opening at as many points as collections of pus may be found: this is done to prevent the possibility of altered pus being pent up. It will be necessary to watch carefully the position of the limb, which the patient will always seek to flex, as a position which gives ease; motion should be absolutely prevented, and light warm emollient applications are then most comfortable. If the limb be much infiltrated, light equable bandaging will be proper, and the patient's strength must be kept up against the exhausting effects of suppuration. *Lond. Med. Gaz.*

---

*Mr. Phillips on Arthritis.*—Acute inflammation of joints, other than those produced by wounds, by gout, and rheumatism, we are also called upon to treat. It may be occasioned by blows, fractures, necrosis, sprains, dislocations, or other violence; by foreign bodies in the joints, by the action of cold, and by many other causes, such as gonorrhœa, parturition, or catheterism; but in these cases, when the inflammation is acute, the synovial apparatus is usually affected. The *symptoms* are very similar to those caused by wounds, and therefore it is unnecessary to recapitulate them here. In some cases the disease has been excessively painful, and has ended in gangrene. (Velpeau.)

When it occurs in persons suffering from gonorrhœa it is less painful, but it is sometimes developed with extreme rapidity; it has occasionally been seen in women after the use of astringent vaginal injections; in either case it often disappears quite as suddenly. It may, however, persist, becoming chronic, and ending in white swelling.

As a consequence of child-bearing, this affection is generally not very painful, neither does it light up severe general reaction; it may be developed quite as suddenly as that from gonorrhœa. Though it usually terminates favourably, it may, like that from gonorrhœa, end in white swelling.

I know of five cases of arthritis following upon catheterism: of these, two died, one recovered, but with anchylosis, the other two recovered, but after long suffering. In one, fever was developed after each attempt to introduce a bougie. The means which I have known to succeed best in gonorrhœal arthritis, are cubebs in large doses, compression, blisters, and purgatives; after, blood-letting and bathing. In these two varieties, mercurial and opium frictions have succeeded well, together with alternative doses of calomel.—*Ibid.*

*Disease of the Cæcum and Appendix Vermiformis, issuing in a Fistulous Opening through the Abdominal Parietes.*—May 15, 1835. Thomas Ryley, aged twenty-two years, weaver, stated that twelve months previous to his admission he received a severe blow on the lower part of the abdomen, to which he attributed his present affliction. It commenced with pain in the abdomen, at first slight, and not incapacitating him from following his employment during the first six months of its existence. From this period it became more violent, and a tumour gradually developed itself on the right side, immediately above Poupart's ligament.

When admitted, the abdomen was very tender, and an obscure tumour could be felt on the right side of the linea alba above the groin; it was very firm, and pressure upon it caused pain. His bowels were moved daily, the fæces being generally light coloured and loose; the swelling continued to extend upwards until the 23d of July, when it burst at the umbilicus, and a considerable quantity of feculent matter of a light colour and thin consistence was discharged; this gave immediate relief to the pain which had accompanied its formation. Fæces were discharged almost daily from the umbilicus, as well as from the anus, the quantity varying in proportion to the violence of the diarrhœa which was present. He died about the middle of September, and, on examination, the following appearances were detected:—

The lower portion of the ileum and the cæcum were extensively thickened in consequence of lymph having been thrown out on some previous occasion. A sinus was found extending from the cæcum to the umbilicus; it was formed by the appendix vermiformis cæci, which was bent upwards and adherent to the anterior parietes of the abdomen, to within about an inch of the umbilicus, with which it communicated by a short fistulous canal. The appendix was thickened and dilated, and the shell of a hazel-nut was impacted in its middle portion. *Ibid.*

---

*On Quinine.* By M. H. Magouty.—An accidental circumstance led M. Magouty to study the action of the ammoniacal salts on quinine at different degrees of temperature, and the following are the results to which he has arrived.

1. That quinine is more soluble in water than is generally imagined, and more so in hot than in cold water.

2. That quinine becomes anhydrous even when in water of the temperature of 60° cent.

3. That quinine can be easily obtained in a crystallized state from its watery solution; but that it is only with difficulty that crystals are got from an alcoholic solution.

4. That ammonia, without the aid of heat, only partially decomposes the salts of quinine, as they do also those of magnesia, but that ammoniacal salts are decomposed by quinine, if aided by a temperature equal to that of boiling water.—*Journ. de Med. Prat. de Bordeaux.*

